# Delegating care as a double-edged sword for quality of nursing care: a qualitative study

**DOI:** 10.1186/s12913-024-11054-4

**Published:** 2024-05-07

**Authors:** Tayebeh Moradi, Mahboubeh Rezaei, Negin Masoudi Alavi

**Affiliations:** Trauma Nursing Research Center, Kashan University of Medical Sciences, Kashan, Iran

**Keywords:** Delegation of care, Insourcing, Outsourcing, Quality of nursing care

## Abstract

**Background:**

Considering the significance of care delegation in enhancing the quality of nursing care and ensuring patient safety, it is imperative to explore nurses’ experiences in this domain. As such, this study aimed to explore the experiences of Iranian nurses regarding the delegation of care.

**Methods:**

This qualitative study was conducted between 2022 and 2023, employing the content analysis method with a conventional approach. The study utilized purposeful sampling method to select qualified participants. Data collection was carried out through in-depth and semi-structured interviews utilizing open-ended questions. The data analysis process followed the steps proposed by Graneheim and Lundman (2004) and involved the use of MAXQDA version 12 software. To ensure the trustworthiness of the data, the study employed the four rigor indices outlined by Lincoln and Guba (1985).

**Results:**

In the present study, a total of 15 interviews were conducted with 12 participants, the majority of whom were women. The age range of the participants fell between 25 and 40 years. Through qualitative data analysis, eight subcategories and three main categories of “insourcing of care”, “outsourcing of care” and “delegating of care to non-professionals” were identified. Additionally, the overarching theme that emerged from the analysis was “delegation of care, a double-edged sword”.

**Conclusions:**

The results of the study revealed that the delegation of care occurred through three distinct avenues: to colleagues within the same unit, to colleagues in other units, and to non-professionals. Delegating care was found to have potential benefits, such as reducing the nursing workload and fostering teamwork. However, it was also observed that in certain instances, delegation was not only unhelpful but also led to missed nursing care. Therefore, it is crucial to adhere to standardized principles when delegating care to ensure the maintenance of high-quality nursing care.

**Supplementary Information:**

The online version contains supplementary material available at 10.1186/s12913-024-11054-4.

## Introduction

Nurses play a crucial role in delivering healthcare services on the frontline. They serve as planners, coordinators, providers, and evaluators of care, performing a wide range of nursing tasks from the moment of admission to discharge to enhance patients’ health and facilitate their recovery [1–3]. Given that nurses constitute the majority of healthcare personnel worldwide, the quality of care they deliver holds significant importance [4]. Hence, any disruptions in the flow of care provided by nurses can lead to a decline in the quality of care [2].

The delegating care or delegation of authority is recognized as one of the factors that impact the quality of nursing care [5]. Various definitions of delegation of authority can be found in the related literature, but they all share a common theme: the transfer of responsibility for an activity from one person to another who accepts the responsibility to carry out the activity in an appropriate manner [6]. Nurses possess the authority to safeguard the health and well-being of each patient, allowing them to assign certain care activities to other qualified and competent individuals [7]. In the 21st century, nurses are expected to possess the ability to delegate care services while also supervising the delegated care. Delegation of care enables nurses to allocate more time to patient care, support, and education [8]. The process of delegating care involves complex decision-making, where nurses must demonstrate leadership and change management skills to establish team cohesion, support patients, and ensure the effective implementation of nursing activities [9]. In essence, delegation is viewed as a leadership skill that can significantly impact the quality of care and patient satisfaction [5]. Campbell et al. (2020) demonstrated that effective delegation of care and proper communication between nurses and nursing assistants can enhance cooperation, job satisfaction, and ultimately reduce negative outcomes for patients [10]. Delegation improves accountability, productivity, and facilitates the development of teamwork [11]. Proper and principled delegation of authority enhances nurses’ knowledge, decision-making abilities, and competence. It also improves communication skills, reduces missed care, and minimizes delays in care [12, 13]. However, the effectiveness of teamwork and delegation between nurses and healthcare assistants varies across different unit cultures and work styles. In some settings, delegation of care may not be executed properly, leading to inefficiencies and decreased effectiveness [14]. Providing safe care is contingent upon safe delegation, and this necessitates that nurses appropriately plan, delegate, and monitor assigned tasks. Failure to delegate care activities safely and appropriately can result in adverse outcomes for patients and contribute to missed nursing care and nursing care rationing [15]. In certain instances, due to high workload, nurses may delegate complex and challenging care tasks to individuals with lower skill levels, and there may be a lack of adequate monitoring of the delegated activities. Consequently, care may be missed, duplicated, or administered improperly [15]. GransjönCraftman’s study highlighted that the high workload experienced by nurses and the challenges they face have led to the delegation of drug administration tasks to unlicensed personnel, potentially compromising the quality of care [16]. Poor delegation practices can result in nurses and nursing assistants working in parallel and separately, rather than functioning as an integrated team. It leads to ineffective communication, inappropriate work dynamics, conflicts between nurses and nursing assistants, and ultimately a decrease in the quality of care [14]. Furthermore, improper delegation of authority can result in non-compliance with care standards, inadequate documentation of provided care, and poor assessment and monitoring of patient conditions [17]. Some nurses lack understanding of the concept of delegation and do not provide proper monitoring when delegating authority. This lack of monitoring also may lead to missed nursing care and potential adverse events for patients [15, 18].

In Iran, the delegation of care is performed by nurses, though in some cases this delegation is inappropriate. The nursing structure in Iran differs from that of other countries. The majority of nurses employed in Iranian hospitals hold a bachelor’s degree and work in hospitals and health centers under the ownership and administration of the Ministry of Health and Medical Education. Nurses in Iran face various challenges such as a shortage of nursing personnel, immediate employment after graduation without prior work experience, overtime work, undefined responsibilities, inadequate equipment, low wages, and a gap between theoretical knowledge and practical application [19, 20]. Consequently, the delegation of care in Iran appears to be distinct from that of other countries and warrants further investigation. Rooddehghan et al. (2015) showed that Iran’s nursing care system lacks specific guidelines for delegating care. As a result, due to the nursing shortage, care is sometimes delegated to non-professionals, including the patient’s family. Additionally, due to high nursing workload, proper monitoring of these delegated tasks is not consistently carried out. Consequently, the provision of nursing care is disrupted, posing a threat to patient safety [8].

Examining the experiences of Iranian nurses regarding the delegation of care can provide valuable insights and a more realistic understanding of their perspectives on this issue. Quantitative research methods may not offer the necessary flexibility and depth required to investigate the experiential aspects of a phenomenon. Therefore, the most suitable approach to explore the experience of care delegation is through the use of qualitative research methods. These methods are particularly effective in exploring questions that involve human interpretations, mentalities, and the depth and complexity of phenomena [21]. Considering the significance of care delegation in the quality of nursing care and the limited research conducted on this topic in Iran, the purpose of this study was to explore the experiences of Iranian nurses regarding the delegation of care.

## Methods

### Design

The main question of this research focused on understanding the experience of Iranian nurses in delegating care. To address this question, the appropriate research method utilized was qualitative research, with conventional content analysis approach. Qualitative content analysis is a research method commonly used to explore individuals’ understanding of everyday life phenomena and interpret the content of qualitative data. In conventional content analysis categories are directly extracted from the data [22].

### Participants and sampling

The participants consisted of nurses working in various units of hospitals in Kashan city, located in the central region of Iran. The inclusion criteria for participating in the study included being a clinical nurse (a nurse who has at least a bachelor’s degree in nursing and is responsible for direct clinical care of the patients), having a minimum work experience of six months, and providing consent to participate. The exclusion criterion was the participant’s decision to leave the research during the study. The participants were selected using purposeful sampling method, taking into account the maximum variation in terms of personal and job characteristics. Sampling continued until data saturation was reached.

### Data collection

After identifying eligible participants and obtaining their consent to participate in the study, information regarding personal characteristics (such as age, gender, level of education, marital status, and number of children) as well as occupational characteristics (including unit of service, work experience, and position) were collected. Data collection was conducted through in-depth and semi-structured interviews utilizing open-ended questions. The first participant was purposefully selected as a key informant among the nurses working in Kashan hospitals, and then subsequent sampling continued with maximum variation. Before the interviews, the participants were contacted by phone to arrange the details. The first researcher introduced herself and explained the purpose of the study to the participants. If the participants agreed to take part, the time and location of the interview were determined based on their preferences and convenience. With consent from the participants, the interviews were recorded using a MP3 Recorder and later transcribed verbatim. The interviews were guided by the following questions: (a) Please explain your experience of delegating care to another person; (b) What kind of care have you delegated to others, and to whom did you delegate the care? (c) What factors influenced your decision to delegate care to others? To encourage the participants and gather more in-depth information, probing questions such as “Please explain more,” “Clarify your statement further,” or “Provide an example” were asked during the interviews. The duration of the interviews ranged from 20 to 70 min. In three cases, due to fatigue of the participants during the interview, it was decided to conduct the rest of the interview in the second session.

### Data analysis

The data analysis commenced after the first interview and continued alongside data collection. This iterative process allowed for a back-and-forth movement between developing concepts and gathering data, enabling subsequent data collection to acquire relevant information to address the main research question [22]. The data analysis procedure followed the steps proposed by Graneheim and Lundman (2004) [23]. The verbatim transcripts of the interviews were imported into MAXQDA software, version 12 for coding and analysis. The researchers thoroughly read the interview texts multiple times to gain a comprehensive understanding. In this research, the entire text of each interview was considered as the unit of analysis. Following that, words, sentences, or paragraphs were identified as meaning units and grouped based on their content and meaning, resulting in condensed meaning units. These condensed meaning units were abstracted, labeled, and transformed into codes. Coding was performed using the participants’ language (in vivo) or based on the researcher’s general understanding of the participants’ speech (in vitro). The codes were then compared to identify similarities and differences and then were categorized into subcategories and main categories, forming the manifest content. Through a process of careful reflection and comparison among the categories, the latent content, which was the theme of this study, was revealed. An example of how data were analyzed is provided in Table 1.

**Table 1:** Examples of meaning unit, condensed meaning unit, and codes

Meaning unit	Condensed meaning unit	Codes
There is a problem with the demand for nurses from other units. There is no control over the work of these nurses and usually, the care by the temporary workforce isn’t done properly.	Lack of control over the work of nurses coming from other units and consequently the reduced quality of nursing care.	- Lack of control over the work of temporary nurses - Doing lower quality of care by temporary nursing staff
Sometimes the unit is so crowded that we’ve to delegate some work to the nurses of the next shift. If a lot of care is left to them, their workload will be increased.	An overcrowded unit forces the nurse to delegate care to the nurses of the next shift. Increased workload of the next shift nurses because of assigning a large number of cares to them.	- Mandatory delegation of care to the next shift nurses due to the overcrowded unit - High workload of the shift due to the number of cares delegated by the previous shift

### Rigor and trustworthiness

In this research, the trustworthiness of the data was assessed using four rigor indices proposed by Lincoln and Guba (1989) [24]. Prolonged engagement with the data for several months, appropriate and long-term participation and interaction with the participants, and member checking increased the credibility of the data. To increase the dependability of the data, immediate transcription of the interviews was performed, followed by seeking the opinions of two expert colleagues in the field of qualitative research (peer Checking). Moreover, the participants themselves reviewed the text of the interviews. For this purpose, several initial interviews, along with the preliminary codes derived from them, were provided to three participants involved in the interviews, allowing them to confirm or suggest corrections. The use of a sufficient and suitable sample with maximum variation in terms of demographic and occupational characteristics strengthened the confirmability of the data. Additionally, to ensure the transferability of the data, direct quotes from the participants were included, and comprehensive details related to the participants were described to provide a rich understanding of the context.

## Results

In the present study, a total of 15 interviews were conducted with 12 eligible participants. The majority of the participants were women whose ages ranged from 25 to 40 years. Additional characteristics of the participants can be found in Table 2.

**Table 2:** Demographic and occupational characteristics of the participants

No.	Gender	Age (year)	Education	Marital status	No. of children	Unit	Work experience (year)	Position	Interview time (minutes)	Number of interviews
1	Female	25	Master’s degree	Married	No child	Internal, orthopedics, rheumatology and neurology	2	Nurse (rotating shift)	25, 30	2
2	Male	36	Bachelor’s degree	Married	1	ICU	13	Nurse (rotating shift)	25, 30	2
3	Female	38	Master’s degree	Married	2	ICU	15	Nurse (rotating shift)	45	1
4	Female	40	Bachelor’s degree	Married	3	Eye & ENT surgery	17	Nurse (rotating shift)	20, 40	2
5	Male	25	Master’s degree	Married	No child	CCU	3	Nurse (rotating shift)	55	1
6	Female	34	Bachelor’s degree	Married	3	Gastrointestinal surgery	13	Nurse (fixed morning shift)	70	1
7	Male	29	Bachelor’s degree	Married	No child	Infectious diseases	6.5	Nurse (fixed morning shift)	45	1
8	Female	26	Bachelor’s degree	Married	1	Pediatric and NICU	2	Nurse (rotating shift)	35	1
9	Female	30	Bachelor’s degree	Married	1	Pediatric	8	Nurse (rotating shift)	30	1
10	Female	27	Bachelor’s degree	Married	No child	Emergency	4	Nurse (rotating shift)	35	1
11	Female	40	Master’s degree	Single	No child	Oncology	16	Nurse (rotating shift)	45	1
12	Female	35	Master’s degree	Married	1	Nursing management office	10	Supervisor	40	1

Qualitative data analysis resulted in the extraction of eight subcategories and three main categories as well as the theme of “delegation of care, a double-edged sword” (Table 3).

**Table 3:** Results of data analysis

Theme	Categories	Subcategories	Examples of codes
Delegation of care, a double-edged sword	Insourcing of care	Seeking assistance from colleagues in the same shift	Seeking assistance from a colleague with a lower workload Seeking assistance from a colleague with more stable patients Seeking assistance from a more experienced colleague
		Seeking assistance from the head nurse	Seeking assistance from the head nurse for changing the dressing of orthopedic patients Seeking assistance fromthe head nurse for perform the care that the nurse is not skilled at Seeking assistance from the head nurse for changing the colostomy bag
		Delegating care to colleagues on the next shift	Delegation of the patient’s dressing to the next shift Delegation of the medication to the next shift
	Outsourcing of care	Patient transfer to other units	Transfer of patients with stable condition to another unit Transfer of patient with unstable condition to another unit
		Demand for extra nursing workforce	Requesting nursing workforce from other units Requesting nursing workforce from the nursing office Requesting for temporary nursing staff
	Delegating of care to non-professionals	Delegating care to nurse assistant	Requesting the nurse assistant to do an electrocardiography Requesting the nurse assistant to perform a gavage Delegating the patient out of bed to the nurse assistant without the nurse’s supervision
		Delegating care to nursing students in the internship course	Delegating drug therapy to an inexperienced student Delegating the preparation of drugs to the inexperienced student Delegating unsupervised oxygen therapy to a nursing student
		Delegating care to the patient’s family	Delegating of gavage to the patient’s family The patient’s change position by the patient’s family Disconnecting and reconnecting the IV drip by patient’s family

After analyzing the data, it was found that nurses had experienced delegation of care in three ways: “insourcing of care”, “outsourcing of care” and “delegating of care to non-professionals”.

### Insourcing of care

One of the categories extracted in this research was insourcing of care. Delegation of the patient’s care by the nurse to their colleagues in the same unit is called insourcing of care. In this type of delegation, the nurse delegates some of her/his patient care to other nurses in the unit for various reasons, including high workload or serious patient conditions. The insourcing of care can be accomplished through three methods: Seeking assistance from colleagues in the same shift, seeking assistance from the head nurse, or delegating care to colleagues on the next shift.

#### Seeking assistance from colleagues in the same shift

One form of the insourcing of care was seeking assistance from colleagues in the same shift. In the interviews, the nurses mentioned that sometimes due to the crowded unit, they asked for help from other nurses in their unit to provide care to their patients. In this regard, one of the participants said:

*“When the unit is crowded, if I have a colleague whose work is less, or whose patients are more stable, or my colleague is more experienced, I get help from them, and they usually help me.” (*Participant No. 2).

Another participant said:

*“Colleagues usually cooperate with me. For instance, a few days ago, the unit was extremely busy, and I had a patient with appendicitis. I asked my colleague to assist by providing the patient with clothes and completing a pre-operative form for them before going to the operating room. Also, she helped me by sending the patient’s test results.*”*(*Participant No. 8).

Indeed, in delegating care to other nurses, the participants pointed out that nursing care is delegated to a colleague who has less workload, otherwise, the delegated care may be missed or overlooked. Two participants commented on this issue as follows:

*“Since other colleagues may also have their own tasks to complete, we typically seek help from a colleague who less busy. This ensures that we can collectively finish all the work while allowing that colleague to assist us as well. Otherwise, our colleague may not be able to accommodate our requests or can inadvertently forget them.” (*Participant No. 4).

*“Sometimes my colleagues delegate care to me, but I forget. For example, I was given an urgent care to do, or to notify sonography; I was so busy or involved in my own tasks that I forgot to do it or follow it up.” (*Participant No. 10).

#### Seeking assistance from the head nurse

Another subcategory of care insourcing was seeking assistance from the head nurse. Some nurses mentioned that when they face a high workload, lack of skill in caring for a specific situation, or when it is not possible to provide care due to moral or religious reasons, and other nurses are unable to help, they turn to the head nurse for assistance in performing the necessary care:

*“Here, the head nurse is a man. If we’re on an all-female shift, we ask him to lend a hand with the urology patient, and he gladly pitches in. Sometimes, he assists us in changing the dressing of orthopedic patients which are big and heavy. We appreciate his help because it allows us to handle the patient’s care more efficiently.” (*Participant No. 6).

*“Sometimes, nurses don’t know how to handle certain care, so they call the head nurse to come and do it, hoping to learn from them. Tasks like changing the colostomy bag, taking care of the port, removing the sheet after angiography, washing the chest bottle, and so on.” (*Participant No. 12).

#### Delegating care to colleagues on the next shift

In certain situations, such as overcrowding of units, patients with serious conditions and prolonged diagnostic and treatment procedures, nurses are compelled to delegate some of the patient care responsibilities for the current shift to the next shift. This practice can lead to delayed care during the next shift, or sometimes an increased workload for the nurses on the next shift. The following are the statements of some of the participants:

*“For example, when a patient goes for dialysis, ultrasound, or needs a simple x-ray, it takes a while for them to return to the unit, and you won’t have enough time to fully take care of them. So, you end up leaving some of the patient’s care for the next shift*.”*(*Participant No. 2).

*“Sometimes, the unit gets super hectic, and the patient is very ill; so that you end up having to pass on some of the tasks to the next shift. But the care gets delayed, and that puts extra pressure on the next shift*.”*(*Participant No. 4).

*“It’s just unfortunate when a very sick patient transfers to your unit and you find out that some of his/her nursing cares weren’t done or were done incompletely, and they’re delegated to your shift. You already have your own work to handle, but now you’ve to deal with the unfinished care from the previous shift as well. It adds to your tasks and makes it hard to complete all your duties for the patient during your shift.” (*Participant No. 3).

### Outsourcing of care

Another category extracted was care outsourcing. Outsourcing of care refers to transferring patient care to other units or delegating certain patient care to temporary nurses from other units. Factors such as high workload, nursing shortage, or time constraints are some reasons for nursing care outsourcing. In such cases, the nurse has to rely on temporary nurses who are transferred to the unit or transfer the patient to another unit.

#### Patient transfer to other units

To ensure the continuation of patient treatment and care, some nurses transferred their patients to other units. These nurses stated that when considering the transfer of a patient to another unit, they first contact the target unit. They then assess the conditions of the destination unit to ensure it meets the requirements for accepting the patient. If the unit meets the necessary conditions, the patient is subsequently transferred to that unit. Verifying the conditions of the destination unit before transferring the patient is crucial to avoid potential risks and ensure timely and appropriate care. Failing to assess the conditions of the destination unit could jeopardize the well-being of the patient and result in delays or even the absence of necessary care.

*“We, in the pediatric unit, call the gynecological surgery unit. If they show us the green light, we send stable patients over five years old there. Another example is when the extra beds were all occupied and we had no choice, we sent patients with more stable conditions to the gynecological surgery unit. It helps to decrease our tasks a bit and ensures we can manage all the cares*.”*(*Participant No. 9).

However, another participant said:

*“Sometimes a patient with “special conditions” such as a CCU patient who also needs monitoring is transferred to our unit because of an infection. Our unit is an infectious disease unit, but sometimes the large number of patients and the workload of the unit means that the nurses cannot provide the full care of them, and some care may be missed.” (Participant No.7).*

#### Demand for extra nursing workforce

Another aspect of care outsourcing involves the demand for a nursing workforce or temporary nursing staff from other units. The nurses emphasized that they often request temporary nursing staff from the nursing management office to assist them with providing care during busy periods of the unit. Two participants expressed the following:

*“Sometimes, when there’s a lot of work and the number of nurses is low, we call the nursing office and ask them to provide us with a temporary nurse*.” (Participant No. 2).

*“If I realize that I won’t be able to finish all the tasks before the end of my shift, I’ll ask the supervisor to provide some temporary nursing help, maybe for an hour or to fully assist us throughout the shift.” (*Participant No. 9).

Of course, the transfer of temporary staff to the unit did not always lead to all the care being done completely, and some care might be omitted or missed in this process:

*“We sometimes ask for temporary staff to come into the unit as well. However, we can’t guarantee that the care will be fully carried out because the staff from other units have different routines. The type of care provided for the patients varies until they become familiar with the unit’s care practices and routines. So, it’s not always possible to complete all of care entirely*.”*(*Participant No. 3).

### Delegating of care to non-professionals

Delegating patient care to individuals who are not qualified is referred to as delegation of care to non-professionals. In situations where nurses face an inappropriate nurse-to-patient ratio and need to provide care in any way possible, they may delegate some of their patient care responsibilities to individuals who lack the necessary qualifications. There are three types of unprincipled delegation of care to non-professionals, namely delegation to a nurse assistant, to a nursing intern, and to the patient’s family.

#### Delegating care to nurse assistant

Nurses mentioned that there are instances when they delegate care to non-professional nurses. Specifically, they highlighted the delegation of care to nurse assistants who may lack the necessary academic knowledge and skills required for performing certain care tasks.

“*Here, we ask the nurse assistants to perform ECG, or BS, or even in some cases do venipuncture.*” (Participant No. 8).

“*When the hospital gets busy, we usually ask the nurse assistant to do tasks like taking an ECG or performing a gavage for the patient, even though they may not be highly skilled in these areas.*” *(*Participant No. 5).

#### Delegating care to nursing students in the internship course

Some nurses mentioned the delegation of care to nursing students in the internship course. While this practice might reduce some of the nurse’s tasks, it is important to note that nursing students are still in the learning process and may not have the ability to independently perform care without the supervision of their instructor. This type of improper delegation of care has the potential to endanger patient safety.

*“Some colleagues delegate the responsibility of medication to interns. Some students, who are still learning nursing care, may not yet have the confidence to administer medication independently without supervision*.”*(*Participant No. 8).

Another participant stated:

*“At times, nurses assign interns to care for patients, but these interns may forget to do some necessary care.” (Participant No. 11).*

#### Delegating care to the patient’s family

Many nurses referred to the delegation of care to the patient’s family. In other words, they occasionally delegate tasks that should be performed by the nurses or other members of the healthcare team to the patient’s families. However, they acknowledged that this practice is not ideal or appropriate.

*“It’s better if patients have someone accompanying them when they are hospitalized because when we’re overwhelmed with work, we often have to rely on the patient’s companion to take care of certain tasks, even though we’re aware it’s not the ideal approach*.” (Participant No. 10).

*“Unfortunately, there are times when nurses pass on certain care tasks to the patient’s family; Tasks such as administering tube feeding, assisting with repositioning and movement, and even providing oxygen therapy. In some cases, the nurse instructs the patient’s companion to disconnect and reconnect the IV drip, disconnect and reconnect the monitoring equipment, or administer insulin to the patient without supervision.”*(Participant No. 12).

### Delegation of care, a double-edged sword

The findings of this study revealed that the overarching theme extracted from the analyzed data was the “delegation of care, a double-edged sword.” Contemplating the key categories derived from the participants’ statements indicated that nurses, due to factors such as inappropriate nurse-to-patient, emergent situations, time constraints, and nursing shortage, delegate a portion of their patient care responsibilities to others. However, this care strategy can have a dual and paradoxical impact on the quality of patient care. On one hand, delegating care can reduce the workload of nurses and enable them to better attend to their other patients. On the other hand, it is associated with potential drawbacks such as delayed care, missed care, neglecting certain aspects of care, or a decrease in the overall quality of nursing care provided.

*“Sometimes, because we’ve so much work to handle, we end up passing on certain care tasks to our colleagues. It helps us manage our patients’ caring tasks, but the downside is that we can’t supervise or ensure that the delegated tasks are done correctly. On the flip side, there are instances when we ask our colleague, “Did you do that?” only to find out that they completely forgot to do it*.” (Participant No. 11).

## Discussion

This study aimed to explore the experiences of Iranian nurses regarding care delegation. It was found that in situations such as the nurses faced a high workload and insufficient staffing, they were compelled to delegate certain patient care responsibilities to others. This delegation took various forms, including delegation of care to colleagues within the same unit (insourcing), colleagues from other units (outsourcing), or non-professional individuals. In certain situations, delegating care can be beneficial as it helps to reduce the nurse’s tasks and promotes teamwork and cooperation. However, there are instances where delegation not only fails to be helpful but also diminishes the quality of care, sometimes resulting in missed or compromised care. Hence, it can be said that the delegation of care acts as a double-edged sword in terms of its impact on the quality of nursing care, with both positive and negative outcomes. Consequently, it becomes crucial to pay close attention to how care is delegated to others to minimize the negative outcomes and enhance the quality of nursing care.

In this study, nurses mentioned delegating care to colleagues within their own unit, which includes other nurses and head nurses. Ghezeljeh et al. (2020) found that increased collaboration among nurses as a result of delegating care can lead to a reduction in missed nursing care [25]. Similarly, Gibbon and Crane (2018) revealed that effective teamwork serves as a strategy to mitigate care left undone [26]. The presence of financial constraints, nursing shortage, and the high complexity of patient care have created an environment where delegation becomes necessary. Proper and standardized delegation of authority plays a crucial role in improving patient care outcomes. To ensure correct delegation of authority, three fundamental components—responsibility, authority, and accountability—must be considered. The nurse should bear the responsibility for patient care, possess the necessary authority and competence to provide care and be accountable for caring for the patient. Based on these three components and considering the five rights of delegation—namely, the right task, right circumstances, right person, right supervision, and right direction and communication—the nurse can appropriately delegate care to another individual. Among these five rights, the right of communication/direction is particularly vital in safeguarding the quality of nursing care and ensuring patient safety [27]. Hence, it is not sufficient to solely delegate care to a colleague. For a successful transfer of care, factors such as effective communication, competence, and knowledge of the receiving nurse, as well as their attitude and duties, must be taken into account [26]. In this case, Rooddehghan et al. (2015) revealed in their study that improper delegation of care can occur due to insufficient staffing and high workload, leading to a lack of proper supervision over delegated care. As a result, there is a possibility that the care assigned to another nurse may not be executed correctly [8].

In this study, nurses reported the delegating of care to the next shift. While delegating care to the next shift may decrease a nurse’s tasks, it also presents challenges such as care delays or an increased workload for the subsequent shift. Campagna et al. (2021) emphasized that nurses often delegate some care tasks to the next shift, resulting in delayed patient care, which can ultimately impact care quality [28]. Similarly, Mantovan et al. (2020) found that nurses tend to delegate care to the next shift when faced with a high workload [29]. Nurses employ strategies such as delaying care or delegating care to the next shift before omitting nursing tasks. However, any delay in the provision of nursing care diminishes its quality and have adverse consequences for the patient [29–31].

In the present study, the nurses identified patient transfer to other units as a significant strategy in delegating care to reduce their workload. Patient transfer within the hospital is an important aspect of patient care management, often employed to improve care provision. However, it is important to recognize that patient transfers can lead to various physiological changes that may have a negative impact on the patient’s prognosis. Therefore, patient transfers should be conducted systematically, following evidence-based guidelines. The decision to transfer a patient should be made after careful consideration of the potential benefits concerning the associated risks. It should be based on the concept of “stabilization and transfer,” starting with stabilizing the patient in the transferring center and ensuring continuity of care until the patient reaches the receiving center [32, 33]. It is pivotal to note that transferring a patient to another unit solely to reduce the workload of the current unit may pose risks to the patient’s health.

One of the strategies mentioned by the nurses was the request for temporary nurses from other units. Studies in this area indicate that employing temporary nursing staff can help diminish the nursing workload. However, it may have adverse economic implications due to the potential increase in negative outcomes for patients. Furthermore, evidence suggests that the presence of supportive nursing staff in a unit might increase the workload for regular nurses. This is because the regular nurses must dedicate more time to supervising these individuals, and they may not be able to provide patient care to the same extent as the regular staff members of the unit [34]. Senek et al. (2020) demonstrated that the use of temporary nurses in a unit leads to an increase in unfinished nursing care [35]. Nursing managers believe that employing temporary nurses to compensate for staff shortages and high workloads in a unit does not necessarily result in an improvement in the quality of services [36].

Nurses also reported instances where they delegate care to non-professionals, such as nursing assistants or nursing students and interns. Chua et al. (2019) found that registered nurses (RNs) delegate certain routine care tasks, such as monitoring vital signs, to enrolled nurses (ENs). However, it is important to note that these nurses may not possess sufficient clinical knowledge regarding monitoring and controlling vital signs [37]. Jasemi et al. (2018) conducted a qualitative study exploring the experiences of nursing students in clinical education. The findings revealed that one of their challenges in clinical education is the delegation of nursing care to them without adequate training and supervision. The students also highlighted that the expectations of the nurses and their mentors are often unreasonable and exceed the students’ capabilities, potentially compromising patient safety [38]. In another study, nursing students expressed a desire for proper guidance and support when performing nursing care [39].

The utilization of nursing assistants or nursing students in place of registered nurses is an example of a skill mix in healthcare settings. Because of the nursing shortage and cost-cutting efforts, some hospitals have adopted a combination of professional nurses (registered nurses) and less skilled staff [40]. However, research demonstrates that the lower the ratio of registered nurses among the nursing staff responsible for direct patient care, the higher the patient mortality rates, poorer quality of care, and increased frequency of adverse outcomes [41, 42]. Aiken et al. (2017) also showed that for every 10% decrease in the proportion of registered nurses compared to the overall nursing staff, there is an 11% increase in the likelihood of patient mortality. Beyond the risk of preventable deaths among patients, increasing the nursing skill mix can have negative results on overall care quality [41].

In the present study, it was observed that nurses delegate certain aspects of care to family members and companions of the patients. Family members play a crucial role in patient care and are considered as caregivers. They can provide valuable support in caring for patients alongside the healthcare team and nursing staff. This highlights the importance of patient- and family-centered care, where the involvement and participation of family members are emphasized. However, the role of the family in the hospital setting is primarily one of support and participation in decision-making, while the caregiver role becomes more prominent after discharge when the patient is at home [43, 44]. Aein et al. (2009) reported that although hospital policies emphasize that nurses should not delegate nursing care tasks to the patient’s family, in certain situations, nurses may delegate some aspects of care to the patient’s family, particularly parents, to reduce their workload. However, this practice has resulted in family dissatisfaction and compromised patient safety [45]. Rooddehghan et al. (2015) also stated that some nurses delegate care responsibilities to the patient’s family or non-professional individuals. It can lead to deficiencies and errors in care, posing a potential threat to the patient’s life [8].

### Strengths and limitations

This study represents the first investigation in Iran that explores nurses’ experiences with delegating care. The findings of this study can enhance the understanding of clinical nurses and nursing managers regarding delegation of care and provide better insights for potential improvements. However, it is important to acknowledge that, as a qualitative study, the generalizability of the findings may be limited to societies with similar backgrounds and cultures.

## Conclusion

The findings of the study revealed that care delegation occurred through three methods: insourcing, outsourcing, and delegation of care to non-professionals. Nurses often resorted to delegating care to others due to reasons such as high workload, time constraints, aiming to prevent delays in care provision. This delegation of care had both positive and negative outcomes. On the one hand, it helped reduce nurses’ workload and fostered team cooperation. On the other hand, it sometimes compromises the quality of care and even leads to missed nursing care. Hence, the act of delegating care can be viewed as a double-edged sword for the quality of nursing care, with the potential for both positive and negative consequences. It is crucial to recognize that care delegation should adhere to correct and standardized principles. Incorrect delegation of care can diminish the quality of nursing care and jeopardize patient safety.

Considering the significance of correct delegation of care and its impact on enhancing nursing care, it is important for nursing managers and policymakers to develop written educational programs for nurses focusing on the proper delegation of care. This will ensure that nurses are familiar with delegation methods and principles, enabling them to effectively manage their workload and ensure comprehensive care. Furthermore, it is essential to incorporate the topic of correct delegation of care into the nursing curriculum, particularly through a nursing management course. By teaching undergraduate nursing students the appropriate methods of delegation, they will be equipped to utilize this approach correctly in their future clinical practice.

### Electronic supplementary material

Below is the link to the electronic supplementary material.

Supplementary Material 1

## Data Availability

No datasets were generated or analysed during the current study.

## Abbreviations

BS: Blood Sugar
ECG: Electrocardiography
MAXQDA: MAX Qualitative Data Analysis
