# Supplementary material for: Delegating care as a double-edged sword for quality of nursing care: a qualitative study
Source: BMC Health Serv Res. 2024 May 7;24:592. doi: 10.1186/s12913-024-11054-4 (PMC11075185; doi:10.1186/s12913-024-11054-4)
Supplement: Supplementary file 1 — Supplementary Material 1 [file 12913_2024_11054_MOESM1_ESM.docx]

**The Researcher Made “Interview Guide”**

**Main Questions:**

a) Please explain your experience of delegating care to another person.

b) What kind of care have you delegated to others, and to whom did you delegate the care?

c) What factors influenced your decision to delegate care to others?

**Probe Questions:**

- Please explain more
- Clarify your statement further
- Provide an example
